# Incidence of, Risk Factors for, and Mortality Associated With Severe Acute Kidney Injury After Gunshot Wound

**DOI:** 10.1001/jamanetworkopen.2019.17254

**Published:** 2019-12-11

**Authors:** Ambarish M. Athavale, Chih-Yuan Fu, Faran Bokhari, Francesco Bajani, Peter Hart

**Affiliations:** 1 Division of Nephrology, John H. Stroger Jr Hospital of Cook County, Chicago, Illinois; 2 Chang Gung Memorial Hospital, Department of Trauma and Emergency Surgery, Chang Gung University, Taoyuan City, Taiwan; 3 Cook County Trauma and Burns Unit, John H. Stroger Jr Hospital of Cook County, Chicago, Illinois; 4 Division of Trauma, John H. Stroger Jr Hospital of Cook County, Chicago, Illinois

## Abstract

**Importance:**

Acute kidney injury increases the risk of mortality in hospitalized patients. However, incidence of severe acute kidney injury (SAKI) and its association with mortality in civilians with gunshot wounds (GSWs) is not known.

**Objective:**

To determine the incidence of and risk factors associated with SAKI and acute kidney injury requiring dialysis (AKI-D) after GSWs and the association of SAKI and AKI-D with mortality among civilians in the United States.

**Design, Setting, and Participants:**

This retrospective cross-sectional study included civilians with GSW reported to the National Trauma Data Bank between July 1, 2010, and June 30, 2015. Torso GSWs were included in study; GSWs to the head were excluded. The data were analyzed between September and November 2018.

**Exposure:**

Civilians with GSW.

**Main Outcomes and Measures:**

Incidence of SAKI and AKI-D; association of SAKI and AKI-D with mortality.

**Results:**

Most of the 64 059 civilian GSWs affected men (57 431 [89.7%]) and racial/ethnic minorities (36 205 [56.5%] African American individuals; 9681 [15.1%] Hispanic individuals). Incidence of SAKI was 2.3% (1450 of 64 059), and incidence of AKI-D was 0.9% (588 of 64 059). On multivariate analysis, SAKI was associated with older age (odds ratio [OR], 1.02; 95% CI, 1.01-1.02; *P* < .001), male sex (OR, 1.37; 95% CI, 1.12-1.66; *P* = .002), diabetes (OR, 1.55; 95% CI, 1.20-2.00; *P* = .001), hypertension (OR, 1.76; 95% CI, 1.46-2.11; *P* < .001), Glasgow Coma Scale score (OR, 0.98; 95% CI, 0.96-0.99; *P* = .002), sepsis (OR, 13.83; 95% CI, 11.77-16.24; *P* < .001), hollow viscus injury (OR, 2.31; 95% CI, 2.05-2.59; *P* < .001), and injury severity score (OR, 1.02; 95% CI, 1.01-1.02; *P* < .001); AKI-D was associated with systolic blood pressure (OR, 0.99; 95% CI, 0.99-1.00; *P* < .001), sepsis (OR, 1.56; 95% CI, 1.18-2.04; *P* = .001), and injury severity score (OR, 1.01; 95% CI, 1.01-1.02; *P* = .001). Mortality was significantly higher in patients with AKI-D (167 of 588 patients [28.4%]) compared with patients with SAKI (172 of 862 [20.0%]) and no SAKI or AKI-D (5521 of 62 609 [8.8%]) (*P* < .001). Mortality was associated with older age (OR, 1.01; 95% CI, 1.01-1.01; *P* < .001), systolic blood pressure (OR, 0.997; 95% CI, 0.997-0.998; *P* < .001), Glasgow Coma Scale score (OR, 0.87; 95% CI, 0.87-0.88; *P* < .001), SAKI (OR, 2.32; 95% CI, 1.93-2.79; *P* < .001), AKI-D (OR, 1.46; 95% CI, 1.12-1.90; *P* < .001), hollow viscus injury (OR, 1.87; 95% CI, 1.76-1.98; *P* < .001), and higher injury severity score (OR, 1.01; 95% CI, 1.01-1.01; *P* < .001). After matching for variables except SAKI or AKI-D, patients with SAKI were twice as likely to die than patients without SAKI (320 of 1391 [23.0%] vs 158 of 1391 [11.4%]; *P* < .001).

**Conclusions and Relevance:**

In this cross-sectional study, SAKI among civilians who experienced GSWs was associated with mortality.

## Key Points

**Question:**

What is the incidence of severe acute kidney injury (SAKI) after gunshot wound (GSW) among civilians in the United States, what risk factors are associated with SAKI, and is SAKI associated with mortality?

**Findings:**

In this cross-sectional study of civilians in the United States who experienced GSWs, the incidence of SAKI was 2.3%, and the incidence of SAKI requiring dialysis was 0.9%. Patients with GSW who developed SAKI were twice as likely to die as those without SAKI.

**Meaning:**

In this study, SAKI was a significant complication after GSW and was associated with mortality.

## Teaser

This cross-sectional study determines the incidence of and risk factors associated with severe acute kidney injury (AKI) and AKI requiring dialysis after gunshot wounds and the association of severe AKI and AKI requiring dialysis with mortality among civilians in the United States.

## Introduction

Firearm-related injury caused 38 658 deaths in United States in 2016.^1^ Civilian gunshot wounds (GSWs) are the cause of approximately 30 000 hospitalizations and 2500 in-hospital deaths every year in the United States.^2^ Survivors of GSWs experience long-term morbidity from injury to various organ systems, such as spinal cord injury,^3^ colonic injury,^4^ and amputation from vascular injury.^5^ Like other organ systems, the urinary tract can be affected by GSW, resulting in acute kidney injury (AKI) from direct injury to the urinary tract or acute tubular necrosis from hypotension, rhabdomyolysis, or sepsis. Acute kidney injury is known to increase the morbidity and mortality of critically ill patients in the intensive care unit setting.^6^ Previous research^7^ has reported 22% all-cause mortality in US military service members after traumatic AKI that required dialysis. However, the incidence of severe AKI (SAKI) and AKI requiring dialysis (AKI-D) after GSW and the association of SAKI and AKI-D with mortality after GSW in the US civilian population have not been studied. In this article, we describe the incidence of SAKI and AKI-D after GSW in civilians in the United States, the incidence and types of urinary tract injury after GSW, the factors associated with SAKI and AKI-D after GSW, and the association of SAKI and AKI-D after GSW with mortality.

## Methods

This is a retrospective cross-sectional study of civilians residing in the United States with GSW reported to the National Trauma Data Bank^8^ (NTDB), a registry of patients, including those with GSWs, admitted to trauma centers in United States. Thus, GSW injuries that resulted in death before hospital arrival were excluded from NTDB, and only patients with GSWs who were transported to trauma centers alive were included in this study. Clinical information is stored in NTDB in deidentified form and available for quality improvement and research. All patients in NTDB with GSW to the torso between July 1, 2010, and June 30, 2015, were included in the study. These dates were chosen to keep the study contemporary and avoid the transition from *International Classification of Diseases, Ninth Revision *(*ICD-9*) to *ICD*-*10*. The data were analyzed between September and November 2018. The study was approved by the Cook County Health institutional review board with exempt status. Because this study analyzed deidentified information from the NTDB, individual patient consent was not required. The authors followed the Strengthening the Reporting of Observational Studies in Epidemiology (STROBE) reporting guideline for observational studies.

The primary aim of the study was to define the incidence of SAKI and AKI-D, which were identified by the trauma centers reporting data to NTDB. We used the AKI code in NTDB to identify patients with AKI. In NTDB, AKI was defined as an increase in serum creatinine levels of at least 300% of baseline, an increase in serum creatinine levels to more than 4 mg/dL (to convert to micromoles per liter, multiply by 88.4), a decrease in estimated globular filtration rate to less than 35 mL/min per 1.73 m^2^ body surface area, reduction of urine output to less than 0.3 mL/kg/h for more than 24 hours, anuria for more than 12 hours, or the need for renal replacement therapy. The definition of AKI in NTDB corresponds to stage 3 AKI according to the Acute Kidney Injury Network (AKIN) classification.^9^ Thus, patients with AKI in NTDB who were included in this study experienced SAKI. We identified AKI-D with *ICD*-*9* codes 39.95, V45.1, V56.0, and V56.1.^10^ We excluded patients with a diagnosis of end-stage renal disease who were receiving dialysis at admission (ie, *ICD*-*9* code 585.6 or codes 39.95, 54.98, V45.1, V56.0, and V56.1 without a diagnosis of AKI). We also excluded patients with missing information and patients with preexisting chronic kidney disease (which was recorded in the NTDB file RDS_COMORBID as COMORKEY = 9). We used the following *ICD*-*9* codes to identify injury to urinary tract: 866.0 for injury to the kidney, 867.2 and 877.3 for injury to the ureter, and 867.0 and 877.1 for injury to the bladder and urethra. Demographic information (ie, age, sex, race/ethnicity) and clinical information (systolic blood pressure, heart rate, respiratory rate, Glasgow Coma Scale score, sepsis, and hollow viscus injury) and injury severity score were obtained from the NTDB. Information on all-cause in-hospital mortality, hospital length of stay, intensive care unit length of stay, and length of ventilation was collected from NTDB. All data reported in the current study represent data from the index hospitalization after GSW.

### Statistical Analysis

For descriptive variables, we described the mean and SD for continuous variables and number and percentage for categorical variables. Incidence of SAKI and AKI-D was calculated by dividing the number of patients with SAKI and AKI-D by the total number of patients with GSWs. We reported the number and percentage of patients with injury to the urinary tract. For comparison of risk factors for SAKI, AKI-D, and mortality, we used the *t* test for continuous variables and the χ^2^ test for categorical variables. A value of *P* < .05 was considered statistically significant, and all tests were 2-tailed. The significant variables in univariate analysis were included in a subsequent multivariate logistic regression model using the enter method. The collinearity analysis was also performed, and multicollinearity was defined as a variance inflation factor value greater than 10. With multivariate logistic regression analysis, independent factors associated with SAKI and AKI-D were identified. The association of SAKI and AKI-D with mortality was also assessed with multivariate logistic regression. Variables that were associated with SAKI were also likely to be associated with increased mortality. To adjust for clinically confounding factors between patients with and without SAKI, a 1:1 propensity score matching analysis was used to evaluate the association of SAKI with all-cause mortality in patients with torso GSWs.^11^ Pairs of these groups of patients with the greedy neighbor approach were constructed. A caliper setting of 0.01 was used. Standardized differences were used to confirm a balanced matching result. The matching result was considered balanced when the SD was less than 0.1^12^ All original files of NTDB with required data were merged and analyzed with R statistical software version 3.3.1 (R Project for Statistical Computing). Excel version 16.13.1 (Microsoft Corp) was used for data entry and to draw associated figures.

## Results

Between July 1, 2010, and June 30, 2015, there were 68 251 patients with torso GSWs in NTDB. Of these, 74 (0.1%) were excluded because they were receiving long-term maintenance dialysis and 4118 (6.0%) were excluded because of missing data. Demographic and clinical variables of the study population are presented in Table 1. Of 64 059 individuals with GSWs, most (57 431 [89.7%]) were men. Racial/ethnic minorities were disproportionately affected (36 205 [56.5%] African American individuals; 9681 [15.1%] Hispanic individuals). Of 64 059 patients included in the analysis, 26 512 (41.4%) were from the South, 12 451 (19.4%) from the West, 11 994 (19.3%) from the Midwest, and 9259 (14.5%) from the Northeast. Geographical region was not reported for 3843 patients (5.9%).

**Table 1.** Characteristics of 64 059 Patients With Torso Gunshot Wounds

Characteristic	No. (%)
Age, mean (SD), y	29.8 (42.4)
Sex	
Men	57 431 (89.7)
Women	6628 (10.3)
Race	
African American	36 205 (56.5)
White	16 328 (25.5)
Asian	503 (0.8)
American Indian	334 (0.5)
Native Hawaiian or Pacific Islander	130 (0.2)
Other or unknown	10 559 (16.5)
Ethnicity	
Hispanic	9681 (15.1)
Comorbidities	
Hypertension	3974 (6.2)
Diabetes	1496 (2.3)
Systolic blood pressure, mean (SD), mm Hg	106.9 (103.9)
Respiratory rate, mean (SD), breaths/min	17.2 (27.3)
Glasgow Coma Scale score, mean (SD)	11.7 (21.7)
Injury severity score, mean (SD)	17.2 (41.0)
Complications	
SAKI	1450 (2.3)
AKI-D	588 (0.9)
Sepsis	953 (1.5)
Injury	
Hollow viscus	26 362 (41.2)
Urinary tract	10 076 (15.5)
Kidney	6358 (63.1)
Ureter	1108 (11.0)
Bladder and urethra	2831 (28.1)
Outcomes	
Mortality	5860 (9.1)
Length of stay, mean (SD), d	
Hospital	10.2 (47.4)
ICU	3.3 (33.2)
Ventilation, mean (SD), d	1.4 (12.7)

Abbreviations: AKI-D, acute kidney injury requiring dialysis; ICU, intensive care unit; SAKI, severe acute kidney injury.

A total of 1450 patients (2.3%) had SAKI, and 588 (0.9%) had AKI-D. The rate of SAKI was higher among African American patients (904 of 36 205 [2.5%]) compared with non-Hispanic white patients (327 of 14 315 [2.3%]) and Hispanic patients (31 of 2013 [1.5%]), but race/ethnicity was not associated with SAKI on multivariate analysis. Patients from the South had a higher rate of SAKI (705 of 27 098 [2.6%]) compared with patients from the Northeast (200 of 9290 [2.2%]), the Midwest (301 of 14 107 [2.1%]), and the West (242 of 12 588 [1.9%]). A total of 10 076 patients (15.5%) had injury to the urinary tract, of whom 6358 (63.1%) had injury to the kidneys, 2831 (28.1%) had injury to bladder and urethra, and 1108 (11.0%) had injury to the ureter. Patients with GSW and injury to the urinary tract had a higher incidence of SAKI and AKI-D than patients with no injury to the urinary tract (SAKI: 483 of 10 076 [4.8%] vs 972 of 53 983 [1.8%]; *P* < .001; AKI-D: 211 of 10 076 [2.1%] vs 378 of 53 983 [0.7%], *P* < .001)

On multivariate regression analysis, SAKI was associated with age (odds ratio [OR], 1.02; 95% CI, 1.01-1.02; *P* < .001), male sex (OR, 1.37; 95% CI, 1.13-1.67; *P* = .002), Glasgow Coma Scale score on admission (OR, 0.98; 95% CI, 0.97-0.99; *P* = .002), diabetes (OR, 1.55; 95% CI, 1.20-2.00; *P* = .001), hypertension (OR, 1.76; 95% CI, 1.46-2.11; *P* < .001), sepsis (OR, 13.83; 95% CI, 11.77-16.24; *P* < .001), hollow viscus injury (OR, 2.31; 95% CI, 2.05-2.59; *P* < .001), urinary tract injury (OR, 2.24; 95 CI, 1.99-2.52; *P* < .001), and injury severity score (OR, 1.02; 95% CI, 1.01-1.02; *P* = .001) (Table 2). Patients with AKI-D were more likely than patients with SAKI who did not need dialysis to have lower mean [SD] systolic blood pressure on admission (99.0 [50.2] mm Hg vs 109.3 [46.5] mm Hg; *P* < .001), sepsis (138 of 588 [22.4%] vs 131 of 862 [15.2%]; *P* < .001), and a higher mean [SD] injury severity score (23.0 [13.9] vs 20.2 [12.7]; *P* < .001). Developing AKI-D was associated with systolic blood pressure (OR, 0.99; 95% CI, 0.99-1.00; *P* < .001), sepsis (OR, 1.56; 95% CI, 1.18-2.04; *P* = .001), and injury severity score (OR, 1.01; 95% CI, 1.01-1.02; *P* = .001).

**Table 2.** Multivariate Logistic Regression Analysis of Independent Risk Factors for Acute Kidney Injury

Variable	OR (95% CI)^a^
Age	1.017 (1.013-1.021)
Male sex	1.370 (1.126-1.668)
Glasgow Coma Scale score	0.981 (0.969-0.993)
Sepsis	13.831 (11.772-16.249)
Hypertension	1.760 (1.466-2.114)
Diabetes	1.553 (1.203-2.005)
Injury	
Urinary tract	2.242 (1.993-2.522)
Hollow viscus	2.305 (2.054-2.588)
Injury severity scale score	1.015 (1.012-1.019)

Abbreviation: OR, odds ratio.

^a^ Adjusted for every other variable using logistic regression analysis. For continuous variables, ORs represent change for each additional 1 unit. All *P* values are <.01.

Overall mortality of the study population was 5860 patients (9.1%). The mean (SD) length of hospital stay was 10.2 (47.4) days, in which the mean (SD) length of ICU stay was 3.3 (33.2) days and mean (SD) length of ventilation was 1.4 (12.7) days. Among patients with no SAKI or AKI-D, 5521 of 62 609 (8.8%) died; among patients with SAKI not requiring dialysis, 172 of 862 (20.0%) died; and among patients with AKI-D, 167 of 588 (28.4%) died (*P* < .001). On multivariate analysis, mortality was associated with older age (OR, 1.01; 95% CI, 1.01-1.01; *P* < .001), systolic blood pressure on admission (OR, 0.997; 95% CI, 0.997-0.998; *P* < .001), Glasgow Coma Scale score (OR, 0.87; 95% CI, 0.87-0.88; *P* < .001), SAKI (OR, 2.32; 95% CI, 1.93-2.79; *P* < .001), AKI-D (OR, 1.46; 95% CI, 1.12-1.90; *P* < .001), hollow viscus injury (OR, 1.87; 95% CI, 1.76-1.98; *P* < .001), and higher injury severity score (OR, 1.01; 95% CI, 1.01-1.01; *P* < .001) (Table 3).

**Table 3.** Multivariate Logistic Regression Analysis of Independent Risk Factors for Mortality

Variable	OR (95% CI)^a^
Age	1.009 (1.007-1.011)
In ED	
SBP	0.997 (0.997-0.998)
GCS score	0.871 (0.866-0.876)
SAKI	2.319 (1.927-2.792)
AKI-D	1.455 (1.116-1.898)
Hollow viscus injury	1.866 (1.758-1.981)
Injury severity scale	1.012 (1.011-1.014)

Abbreviations: AKI-D, acute kidney injury requiring dialysis; ED, emergency department; GCS, Glasgow Coma Scale; OR, odds ratio; SAKI, severe acute kidney injury; SBP, systolic blood pressure.

^a^ Adjusted for every other variable using logistic regression analysis. For continuous variables, ORs represent change for each additional 1 unit. All *P* values are <.01.

To identify the excess risk of mortality in patients with GSW who developed SAKI, we matched patients with and without SAKI by age, sex, systolic blood pressure, Glasgow Coma Scale score, sepsis, hypertension, diabetes, urinary tract injury, hollow viscus injury, and injury severity scale score. After matching for these variables, patients who developed SAKI were twice as likely to die as patients who did not develop SAKI (320 of 1391 [23.0%] vs 158 of 1391 [11.4%]; *P* < .001) (Table 4). Patients with SAKI also had significantly higher mean (SD) hospital length of stay (30.7 [28.0] days vs 15.4 [20.3] days; *P* < .001), mean (SD) ICU length of stay (18.5 [19.0] days vs 5.4 [11.0] days; *P* < .001), and mean (SD) length of ventilation (12.5 [15.9] days vs 3.5 [9.8] days; *P* < .001) (Table 4).

**Table 4.** Characteristics of 64 059 Patients With Torso Gunshot Wound With and Without SAKI

Characteristic	No. (%)					
	Before Propensity Matching			After Propensity Matching		
	SAKI Positive (n = 1450)	SAKI Negative (n = 62 609)	Standardized Difference^a^	SAKI Positive (n = 1391)	SAKI Negative (n = 1391)	Standardized Difference^a^
Age, mean (SD), y	34.7 (15.9)	29.6 (14.6)	0.347	34.4 (15.9)	33.7 (15.8)	0.045
Men	1332 (91.9)	56 009 (89.6)	0.149	1278 (91.9)	1290 (92.7)	0.067
In ED, mean (SD)						
SBP, mm Hg	105.1 (48.3)	107.0 (52.5)	0.035	105.2 (48.7)	106.7 (52.2)	0.031
GCS score	11.3 (5.3)	11.7 (5.7)	0.085	11.3 (5.3)	11.4 (5.4)	0.019
Sepsis	263 (18.1)	690 (1.1)	1.648	204 (14.7)	181 (13.0)	0.077
Hypertension	207 (14.3)	3767 (6.0)	0.527	194 (13.9)	174 (12.5)	0.069
Diabetes	92 (6.3)	1404 (2.3)	0.597	80 (5.8)	88 (6.3)	0.056
Injury						
Urinary tract	487 (33.6)	9589 (15.3)	0.566	446 (32.1)	469 (33.7)	0.041
Hollow viscus	967 (66.7)	25 395 (40.6)	0.593	913 (65.6)	962 (69.2)	0.089
ISS, mean (SD)	21.3 (13.3)	17.1 (14.5)	0.290	21.0 (13.1)	21.6 (16.2)	0.042
Outcomes						
Mortality	339 (23.4)	5521 (8.8)	0.634	320 (23.0)	158 (11.4)	0.467
LOS, mean (SD), d						
Hospital	31.2 (28.5)	9.7 (12.7)	1.621	30.7 (28.0)	15.4 (20.3)	0.627
ICU	18.8 (19.1)	3.0 (7.8)	1.939	18.5 (19.0)	5.4 (11.0)	0.846
Ventilation, mean (SD), d	12.8 (16.1)	1.2 (5.8)	1.854	12.5 (15.9)	3.5 (9.8)	0.682

Abbreviations: ED, emergency department; GCS, Glasgow Coma Scale; ICU, intensive care unit; ISS, injury severity score; LOS, length of stay; SAKI, severe acute kidney injury; SBP, systolic blood pressure.

^a^ Standardized difference of at least 0.1 represents significant differences in covariables between groups.

## Discussion

In this retrospective analysis of civilians with GSWs in United States, the incidence of SAKI was 2.3%, and the incidence of AKI-D was 0.9%. To our knowledge, this is the first study to report the incidence of SAKI and AKI-D after a GSW injury in civilians in the United States. Prior studies have reported an AKI incidence of 1.3% in civilian patients with renal trauma, but they included patients with all types of trauma.^13^ Compared with an SAKI incidence of 2.3% in GSWs in civilians in the United States, incidence of SAKI in military personnel with burns was reported as 6.3% according to AKIN criteria.^14^ In a 2019 systematic review of AKI among patients with trauma,^15^ the pooled incidence of AKI was 20.4%, and the pooled incidence of SAKI was 2.85%. Similar to previous reports of civilian GSWs, most GSWs in this study affected men (89.7%), and racial/ethnic minorities were disproportionately represented (56.5% African American and 15.1% Hispanic individuals).^2^

In the current study, 15.5% of patients had penetrating injury to the urinary tract, and kidney was the most commonly injured organ (63.1%), followed by bladder and urethra (28.1%). Overall, 11.0% had ureteral injury. In a study of battlefield wounds during the war in Croatia, the rate of penetrating urinary tract injury was only 2.6%, with kidney injury predominating (45.1%), followed by bladder (16.5%) and ureter (7.8%).^16^ The rate of penetrating injury to the kidney (9.9%) in the current study was also higher than the 5.7% previously reported rate of kidney injury in penetrating trauma.^17^

Acute kidney injury following trauma is thought to be because of acute tubular necrosis from ischemia, sepsis, or rhabdomyolysis. Sepsis, hollow viscus injury, Glasgow Coma Scale score, and injury severity score were associated with SAKI. Patients in the current study who developed AKI-D had lower systolic blood pressure, higher incidence of sepsis, and a higher injury severity scale score than patients with AKI not requiring dialysis. Similar findings have been reported in previous studies of AKI in patients who underwent trauma.^15,18^ This suggests that acute tubular necrosis owing to ischemia or sepsis was likely the main etiology of AKI in this population.

Acute kidney injury was associated with a significant increase in mortality in the current study. Mortality in military personnel with AKI-D was reported to be 22%,^7^ compared with 28.4% among civilians with GSW and AKI-D in this study. Patients with SAKI were twice as likely to die as patients who did not have SAKI, and patients with AKI-D were 3.2 times more likely to die than patients with no SAKI or AKI-D. This suggests that the severity of AKI is associated with increased mortality in patients with GSW. Acute kidney injury is known to increase the risk of mortality among critically ill patients in the intensive care unit.^6^ Chertow et al^19^ reported that an increase of creatinine by 0.5 mg/dL or more increased the odds of mortality 6.5 times and resulted in a mean 3.5-day increase in hospital stay. It is now well recognized that even modest increases in creatinine levels increase the risk of mortality and are associated with subsequent development of chronic kidney disease.^20^ To emphasize the importance of early diagnosis of AKI, the AKIN and Kidney Disease Improving Global Outcomes (KDIGO) propose an increase in serum creatinine level of 0.3 mg/dL within 48 hours as diagnostic of AKI.^9,21^ However, the American College of Surgeons Committee on Trauma defines AKI as an increase in serum creatinine levels of 3.5 mg/dL or greater, and this definition has been used to define AKI in NTDB.^22,23^ Thus, this definition is likely to miss all but the most severe cases of AKI in civilians with GSW. Costantini et al^23^ reported that applying the American College of Surgeons Committee on Trauma definition identified only 3% of patients with AKI compared with the AKIN definition, which identified 30% of patients. There is no specific treatment to reverse AKI, and preventing further injury to the kidney by judicious administration of fluids, use of vasopressors as needed to maintain kidney perfusion, and withdrawal or dose adjustment of nephrotoxic medications is the standard of care. Studies suggest that late nephrology referral may lead to increased mortality and longer duration of dialysis in patients with AKI.^24,25^ Thus, prompt recognition of AKI and early nephrology consultation after GSW may result in better outcomes, and alignment of surgical guidelines for AKI with AKIN and KDIGO guidelines is needed.

### Limitations

This study has limitations. The NTDB is a convenience sample and thus subject to selection bias, inconsistent measurement of variables, quality of care differences among reporting hospitals, and missing data. However, the outcomes of interest in this study (SAKI, AKI-D, and mortality) were hard clinical end points and less likely to be affected by subjective interpretation. Because this is an observational study, residual confounding is a limitation; however, we have included known variables associated with AKI in this study. Only 6% of the eligible study population was excluded because of missing data, and we feel that the present study was representative of population of civilians with GSWs in the United States.

Acute kidney injury is known to increase future risk of chronic kidney disease. However, the available data did not allow us to estimate risk of chronic kidney disease in patients with GSW who developed AKI, and this needs further research. A total of 588 patients required dialysis in this study; however, we do not have information on the actual indications for dialysis (eg, hyperkalemia, uremia, metabolic acidosis volume overload), dialysis modality (eg, intermitted hemodialysis, continuous renal replacement therapy, slow low-efficiency dialysis), or the association of timing of dialysis (ie, early vs late) with outcomes. A prospective study of patients with AKI after GSW would be able to answer these questions.

## Conclusions

In summary, SAKI is a significant problem in civilian patients with GSW and is associated with in-hospital morbidity and mortality. Alignment of surgical guidelines for AKI with AKIN and KDIGO criteria for diagnosis of AKI may result in earlier recognition and management of AKI and possibly lead to better outcomes.
